# Improving the quality of care for mothers, newborns, and children in ten hospitals of the Republic of Tajikistan

**DOI:** 10.7189/jogh.16.04063

**Published:** 2026-02-13

**Authors:** Shoira Yusupova, Gulnora Rasulova, Firuza Zakirova, Zarina Ibragimova, Zamira Abdulloeva, Rakhmatullo Shabanov, Dilrabo Yunusova, Obidjon Aminov, Tinatin Gagua, Khatuna Lomauri, Bayan Babaeva, Sophie Jullien, Oleg Kuzmenko, Nurshaim Tilenbaeva, Aigul Kuttumuratova, Martin Willi Weber, Tajikistan study group for improvement of quality of care for mothers, newborn and children, Gulnora Rasulova, Rano Alieva, Parvina Kurbanova, Surayo Rajabova, Bahar Mirzabekova, Firuza Zakirova, Mavjuda Isaeva, Shamshod Muzaffarrov, Zarina Ibragimova, Zarina Rustamova, Jamila Murodova, Anvar Meniqulov, Rakhmatullo Shabonov, Iftikhor Mirzoda, Jurabek Ishmirzoev, Shuhrat Zubaidzoda, Shamsiya Narzulloeva, Shahriya Safieva, Rajabgul Khalilova, Tinatin Gagua, Tatiana Caraus, Tatiana Calancea, Marina Taratina, Dmitry Babich, Ksenia Gorina, Anastasia Nikonets

**Affiliations:** 1 World Health Organization Country Office of Tajikistan, Dushanbe, Tajikistan; 2 Department of Pregnancy Pathology, National Research Centre for Obstetrics, Gynecology and Perinatology of the Republic of Tajikistan, Dushanbe, Tajikistan; 3 Department of NICU, National Scientific and Research Centre for Obstetrics, Gynecology and Perinatology of the Republic of Tajikistan, Dushanbe, Tajikistan; 4 The National Association of Midwives, Dushanbe, Tajikistan; 5 Soghd Oblast Maternity, Khujand, Tajikistan; 6 Mother and Child Department, Istiqlol Republican Clinical Centre, Dushanbe, Tajikistan; 7 Department of Safe Motherhood and Family Planning, Ministry of Health and Social Protection, Dushanbe, Tajikistan; 8 Department of Child and Adolescent Health and Parenting Skills, Ministry of Health and Social Protection, Dushanbe, Tajikistan; 9 Davit Tvildiani Medical University, Tbilisi, Georgia; 10 Department of Neonatology, Tbilisi, Georgia; 11 National Centre of Medical Education, Astana, Kazakhstan; 12 World Health Organization Regional Office for Europe, Division of Country Health Policies and Systems, Policy and Governance in Health Unit, Copenhagen, Denmark; 13 World Health Organization Regional Office for Europe, Office for Quality of Care and Patient Safety, Athens, Greece; 14 World Health Organization Regional Office for Europe, World Health Organization European Centre for Primary Health Care, Almaty, Kazakhstan

## Abstract

**Background:**

Tajikistan has accomplished reductions in maternal, newborn, and child mortality over the past decades through targeted policies and interventions. Challenges remain in providing quality healthcare due to limited resources, geographic barriers, and inadequate infrastructure. We aimed to evaluate the impact of a quality improvement (QI) initiative implemented in ten district hospitals from 2021 to 2024 to improve maternal, newborn, and childcare to accelerate progress towards achieving the Sustainable Development Goals.

**Methods:**

A baseline assessment was conducted in 2021, with an endline assessment in 2023, using updated WHO quality assessment tools. A multidisciplinary team of national and international experts evaluated hospital performance across three domains: support systems, clinical management, and organisation of care. Data was collected through observations, interviews, and medical record reviews. Project interventions included working with hospital-level Quality Improvement committees, capacity building in effective perinatal care, and the use of the WHO Pocketbook of Hospital Care for Children. Regular supportive supervision and half-yearly collaborative quality improvement meetings were held among the ten hospitals. We graphically displayed, analysed, and summed the assessment scores.

**Results:**

We observed notable improvements in the quality of hospital care, with most facilities progressing from substandard to better-performing categories. Seven out of ten hospitals demonstrated advancements in their maternity and neonatal units, with improvements in clinical management and hospital support systems, including access to drugs and equipment. Challenges remained in paediatric care, with only two of ten hospitals showing improvements in infrastructure and laboratory services, and none improving drug availability. Improvements in infection prevention and control were minimal; however, four in ten hospitals managed to improve their practices despite challenges with resource availability, infrastructure, and current protocols.

**Conclusions:**

Comprehensive QI interventions can raise standards of care in resource-limited settings like Tajikistan. Despite measurable progress, systemic barriers persist, with weak infrastructure, unstable workforce, and limited infection prevention and control, requiring targeted investment and political commitment. Sustained success depends on equitable resource allocation, robust monitoring systems, and the promotion of a non-punitive, systems-oriented culture. Scaling up this initiative nationwide is critical to achieving long-term improvements in health.

Over the past decades, Tajikistan has demonstrated a strong commitment to improving neonatal and child health. The implementation of targeted policies and measures has led to significant achievements in reducing neonatal and child mortality rates. Maternal mortality has improved 3-fold, decreasing from 67.5 per 100 000 live births in 2000 to 20 per 100 000 live births in 2020 [1]. Similarly, under-five child mortality reduced from 43 per 1000 live births in 2010 to 32 per 1000 live births in 2020, and during the same period, the neonatal mortality rate decreased from 20 per 1000 live births to 14 per 1000 live births [2].

Despite the progress, Tajikistan continues to face significant challenges in providing quality healthcare to its growing population. Limited government budget allocations for health remain a major constraint, compounded by geographic remoteness and inadequate transportation systems that hinder access to essential services. Many health facilities continue to operate with outdated or insufficient medical equipment, and a shortage of trained health workers, especially in rural areas, remains a constant barrier to progress [3,4]. Rural healthcare facilities lack diagnostic tools, limiting the early detection of conditions such as pre-eclampsia, which is the leading cause of maternal mortality, followed by inadequate newborn transportation, equipment, or staff [3]. Despite the adoption of the Children's Rights Charters, inadequate clinical protocols and insufficient staff training continue to hinder access to healthcare. Although child protection policies exist, staff training and monitoring systems still require more attention, including pain management and rights awareness [5].

Out-of-pocket payments account for 64% of total health spending, making it one of the highest in the region, leaving households facing catastrophic health expenses, with outpatient medicines and inpatient care as the main cost drivers [4]. According to a health labour market analysis, Tajikistan experiences the highest turnover rate among medical staff, highlighting substantial inequities in the distribution of healthcare workers between rural and urban regions. Nurses manage 63.8% of primary healthcare (PHC) facilities, placing additional strains on health services and highlighting the need to strengthen trust in PHC [6].

Tajikistan, a Central Asian country with a population of over 10 million and an area of 141 400 km^2^, is predominantly mountainous, with 93% of its terrain at high elevations. Tajikistan has the highest population growth in the region (**Figure 1**).

**Figure 1:**
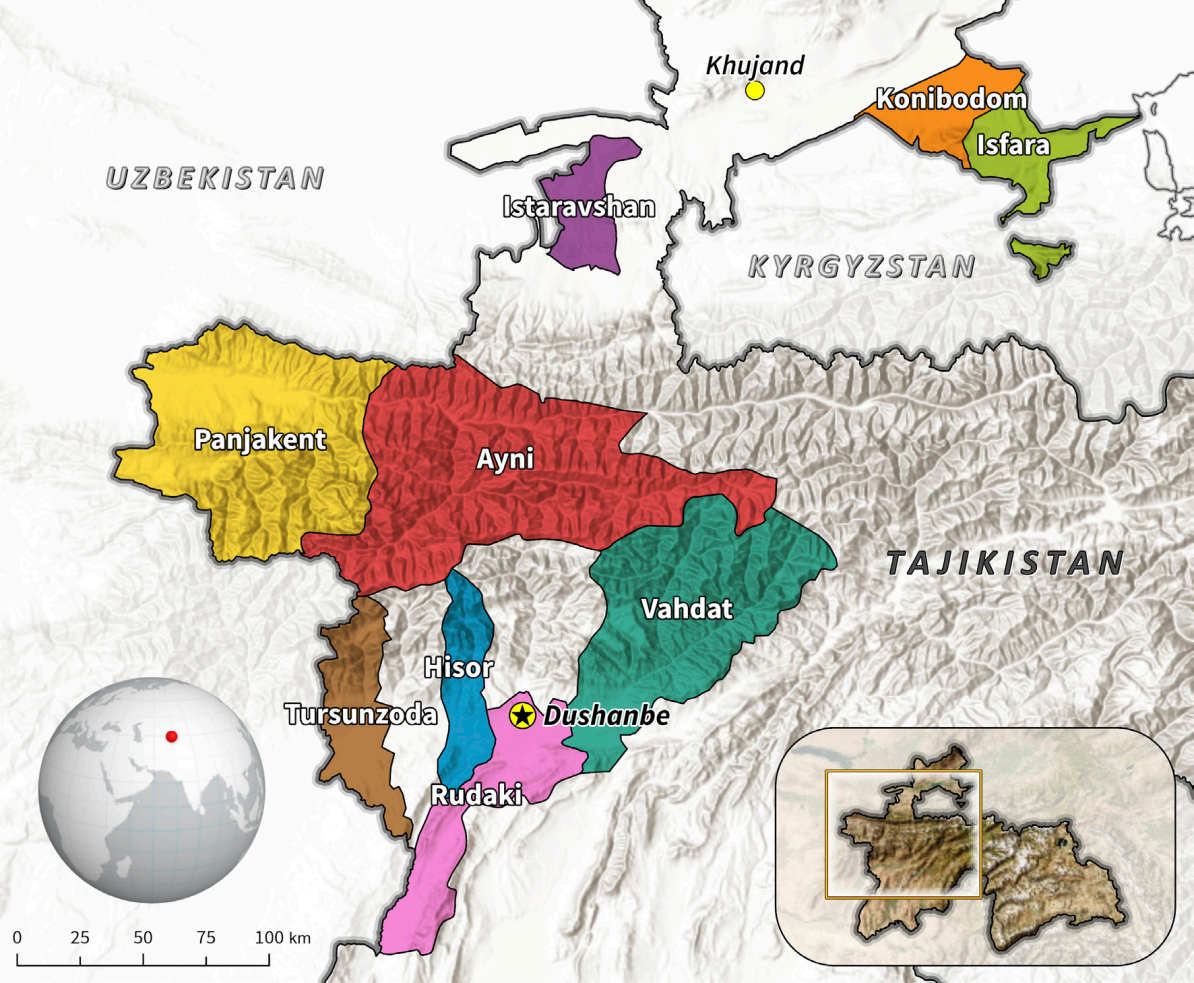
Map of the Republic of Tajikistan with pilot project sites

To address these problems in healthcare delivery and to improve the quality of care for mothers and children, the project ‘Improving the Quality of Hospital Care to Reduce Maternal, Newborn and Child Death and Accelerate the Achievement of Sustainable Development Goal Health Targets’ was implemented in ten intervention hospitals from 2021 to 2024. This was part of a wider project that included Kyrgyzstan, Mongolia, and Vietnam, with a similar approach being taken in Tajikistan. The project targeted district central hospitals and aimed to strengthen the health system by providing quality hospital care to reduce maternal, newborn, and child deaths.

We aimed to analyse the implementation of a complex set of quality improvement (QI) interventions and their outcomes, focusing on their effectiveness in enhancing the quality of hospital care for mothers, newborns, and children [7]. Cross-country comparisons between the two Central Asian countries in this area, as well as findings from the parallel project in Kyrgyzstan, are discussed in separate papers [8−11].

## METHODS

A baseline assessment was conducted in 2021, followed by an endline assessment in 2023. Data were collected using standardised evaluation tools that measured various indicators of obstetric-gynaecological, neonatal, and paediatric quality of care [12,13]. These indicators encompassed clinical practices, the availability of medical equipment, staff qualifications, and patient outcomes.

### Assessment of the quality of hospital care

The primary methods used were the 2015 Hospital care for children: quality assessment and improvement tool and the 2014 Hospital care for mothers and newborn quality assessment and improvement tool [12,13]. The tools used a scoring system derived from expert assessments in three main areas: obstetric-gynaecological, neonatal, and paediatric care. A multidisciplinary national and international team of experienced specialists: obstetricians- gynaecologists, neonatologists, paediatricians, midwives, and paediatric nurses was trained and formed. The three main assessment components were: hospital support system, management of patients with common diseases, and hospital policies and service delivery. Experts also conducted interviews with healthcare workers, patients, and caregivers, performed direct observations, and reviewed medical records, ensuring triangulation in data collection.

### Scoring system

Each clinical practice was rated on a four-point scale from 0 to 3. These categorical scores were then averaged within areas of care to produce summary scores with decimal values (*e.g. 1*.6, 2.3), allowing for more detailed comparisons of performance between hospitals and across assessment periods. Three points indicated that the cate is provided in accordance with international standards (no improvements needed, or only minimal changes required). A score of two denoted a suboptimal level of care, no significant risk to patient health, and patients' rights are generally respected (some improvements needed). One point indicated inadequate level of care, significant risks to patient health, serious violations of the rights of women and newborns (*e.g.* delays or failure to use evidence-based interventions), requiring significant improvements. Lastly, a score of zero indicated very poor quality of care, systematic and serious risks to patient health identified (*e.g.* systematic use of potentially dangerous interventions or lack of resources, essential equipment, and supplies for key procedures such as emergency caesarean (C)-sections, blood transfusions, neonatal resuscitation – a thorough review of the specific area of care is required, and the institution needs substantial improvement.

### Training of assessors and standardisation

The international team trained a multidisciplinary national team selected by the Ministry of Health on effective perinatal care and the use of the World Health Organization (WHO) Pocket Book for hospital care of children under five [14,15], followed by training on the use of the assessment tools of hospital care for maternal, newborn, and child health [12,13].

### Hospital selection

The study was conducted in ten district hospitals in the Republic of Tajikistan, selected jointly by the Ministry of Health of Tajikistan and WHO. Based on their ability to provide inpatient maternal, newborn, and child healthcare, their geographical location, and their focus on areas where minimal other health projects were already under way, to allow for the critical measurement of project interventions and their impact. Four out of the ten hospitals had the capacity to provide tertiary care. The following health facilities were selected: Oblast Maternity Hospital and Oblast Children's Hospital, based in Khujand, and the central district hospitals of Istaravshan, Isfara, Konibodom, Panjakent, Ayni, Rudaki, Hisor, Tursunzoda, and Vahdat.

### Data collection and management

Following the scoring, results were entered into a centralised database and aggregated by domain. To support interpretation and rapid visualisation, a colour-coded categorisation was applied to the average scores, using the following thresholds: red (0–1 score), indicating poor performance and a critical need for improvement; yellow (1.1–1.7 score), indicating below-average performance with significant room for enhancement; amber (1.8–2.4 score), reflecting moderate performance with some deficiencies; and green (2.5–3 score), representing high-quality performance meeting the established standards.

These thresholds enabled a visual representation of each hospital's performance and facilitated the identification of areas requiring immediate attention during facility and national feedback.

Both the baseline (2021) and endline (2023) assessments used the same scoring and visualisation approach. Assessors were blinded to baseline scores when conducting the endline evaluation. Results were compared across the two time points, and changes in performance were categorised as improved (score increase of >0.2 points), no change (score change between −0.2 and 0.2), and deteriorated (score decrease of >0.2 points).

The structured scoring and interpretation method allowed for consistent evaluation, easy visualisation, and meaningful tracking of improvements over time. Differences were calculated and colour-coded, with arrows indicating the trend.

The results of baseline and endline assessments were presented across the three main domains that characterise the quality of obstetric, neonatal, and paediatric care: hospital support systems, clinical management, and organisation of care. For selected indicators, we applied the Wilcoxon signed-rank test to compare baseline and endline scores. A *P*-value of <0.05 was considered statistically significant.

### Project interventions

**Figure 2:**
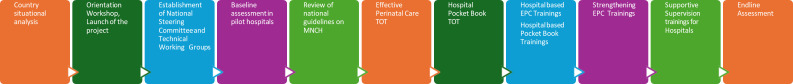
Timeline of assessment and interventions for quality improvement.

Following the baseline assessment, a set of interventions and measures was designed for each hospital in collaboration with the assessment team and hospital staff [8]. Interventions to improve inpatient care were developed jointly with key personnel at each medical institution. QI committees at the facility were either revived or established to track and monitor QI implementation progress. A national feedback meeting, summarising findings across hospitals, examined cross-cutting issues and the need for national action (**Figure 2**).

Obstetricians, gynaecologists, neonatologists, and midwives attended training-of-trainers on effective perinatal care [14], while paediatricians and paediatric nurses were trained to use the WHO Hospital Pocketbook for hospital care for children under five [15]. Capacity building for national specialists and pilot hospital staff in supportive supervision was provided, focusing on updating their knowledge and skills in tracking progress in maternal, neonatal and paediatric care. Each hospital received practical sessions from joint teams of international and national experts.

The National Project Steering Committee, chaired by the Ministry of Health, convened every six months to oversee project progress. Throughout the project period, five half-yearly quality QI workshops were held, bringing together staff from the ten hospitals, including obstetricians, midwives, paediatricians, paediatric nurses and hospital directors, to review progress, share experiences, report progress and plan activities for the next six months. These QI workshops fostered peer learning and collaborative problem-solving as hospitals jointly addressed common challenges. Most hospitals reported common problems, including staff shortages and high staff turnover, poor internal data management and analysis, and a shortage of equipment and resources. Each workshop included formal training on QI tools, such as root cause analysis and the use of run charts for monitoring progress. Within the project, along with supportive supervision, over 1200 health workers were trained to deliver quality maternal, newborn, and child hospital care.

## RESULTS

**Figure 3:**
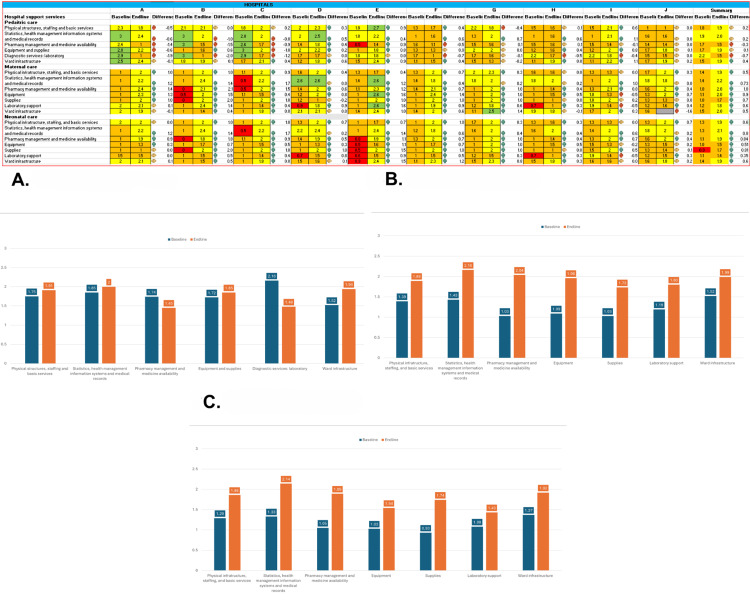
Heat chart of baseline and endline assessment for hospital support services. **Panel A.** Paediatric care. **Panel B.** Maternal care. **Panel C.** Neonatal care.

Overall, the comparative baseline and endline analysis revealed improvements in the quality of hospital care across the ten pilot hospitals. For all three technical areas, hospitals advanced from baseline 1.4 to 1.8 for hospital care and 1.4 to 2.0 for case management and organisation of care (**Figure 3**, Figures S1 and S2 in the **Online Supplementary Document**).

### Hospital support system

Improvement was noted in both maternal care (baseline mean (x̄) = 1.2 *vs.* endline x̄ = 1.9; *P* = 0.01) and neonatal care (baseline x̄ = 1.2 *vs.* endline x̄ = 1.8; *P* < 0.001), demonstrating considerable progress. In contrast, paediatric care showed little measurable improvement (baseline x̄ = 1.8 *vs.* endline x̄ = 1.9; *P* = 0.143). Four out of ten hospitals improved their hospital support systems in both maternal (baseline x̄ = 0.9 *vs.* endline x̄ = 1.9) and neonatal (baseline x̄ = 0.8 *vs.* endline x̄ = 1.8) wards (**Figure 3**).

In the paediatric hospital support system [10], improvements were observed in the physical structure, staffing, basic services, ward infrastructure, and medical statistics (baseline x̄ = 1.7 *vs.* endline x̄ = 1.9). Availability of medicines (baseline x̄ = 1.7 *vs.* endline x̄ = 1.5) and laboratory services (baseline x̄ = 2.2 *vs.* endline x̄ = 1.5) slightly declined (**Figure 3**, Panel A).

For the maternities [9], assessed hospitals improved their hospital support systems across all areas (baseline x̄ = 1.2 *vs.* endline x̄ = 1.9; *P* = 0.01), mainly in the provision and availability of medicines and the supply of equipment (**Figure 3**, Panel B). Nine out of ten hospitals improved their performance in pharmacy management and equipment supply (baseline x̄ = 1.0 *vs.* endline x̄ = 2.0).

Neonatal indicators improved in all ten assessed hospitals for hospital support systems [9]. Improvements were achieved in managing statistics, the availability of medicines, supplies, and drugs (**Figure 3**, Panel C).

### Case management in paediatric care

Overall, case management improved for most indicators [10], with an average improvement score of 0.6 between baseline and endline assessments. Improvements were noticed for several aspects of paediatric care, including acute conditions, emergency treatment, chronic disease management, and follow-up. The quality of triage and emergency care (baseline x̄ = 0.8 *vs.* endline x̄ = 1.9; *P* = 0.005) increased. Improvements were noted in paediatric patient care for common conditions, such as respiratory disease management (baseline x̄ = 1.5 *vs.* endline x̄ = 2.0) and diarrhoea (baseline x̄ = 1.4 *vs.* endline x̄ = 2.0). The management of pneumonia complications and oxygen therapy had positive but not statistically significant changes (baseline x̄ = 1.6 *vs.* endline x̄ = 2.1; *P* = 0.41). Improvements were found in the appropriate use of antibiotics and admission criteria (baseline x̄ = 1.3 *vs.* endline x̄ = 1.9) (Figure S1, Panel A, Subpanels A–C in the **Online Supplementary Document**).

### Newborn care

Hospitals demonstrated notable improvements across various aspects of routine newborn care and the care of the sick newborn [19]. Routine newborn care improved significantly (baseline x̄ = 1.3 *vs.* endline x̄ = 1.8; *P* = 0.007), with notable progress in monitoring and follow-up (*P* = 0.005). Only one out of ten hospitals increased its newborn care indicators (baseline x̄ = 1.1 *vs.* endline x̄ = 1.9). Advanced newborn care increased but not significantly (baseline x̄ = 1.2 *vs.* endline x̄ = 1.6; *P* = 0.144). One hospital showed no improvement in providing adequate advanced newborn care. Most progress was seen in specific areas such as care of preterm and low birth weight infants with all ten hospitals performing better by the endline assessment (baseline x̄ = 1.2 *vs.* endline x̄ = 1.9), and care of sick babies, where improvements were demonstrated in seven hospitals out of ten (baseline x̄ = 1.3 *vs.* endline x̄ = 1.9) (Figure S1, Panel B, Subpanel A in the **Online Supplementary Document**).

### Obstetric care

Results for obstetric care are presented for the three main domains: normal delivery, C-section, and management of obstetric complications and emergency care [8]. Significant improvements were noted in management of normal delivery (baseline x̄ = 1.6 *vs.* endline x̄ = 2.2; *P* = 0.01) and in monitoring indicators (1.7 *vs.* 2.2, *P* = 0.01). Care for C-section improved modestly (baseline x̄ = 1.7 *vs.* endline x̄ = 2.1, *P* = 0.12), while management of obstetric complications showed a significant increase (baseline x̄ = 1.5 *vs.* endline x̄ = 1.9; *P* = 0.02). The main improvement was observed in the management of the first stage of labour (baseline x̄ = 1.5 *vs.* endline x̄ = 2.2) and the third stage of childbirth (baseline x̄ = 1.9 *vs.* endline x̄ = 2.4). The management of C-section care showed considerable improvements in several key areas between baseline and endline assessments. Compliance of C-section standards with international recommendations showed considerable improvement (baseline x̄ = 0.25 *vs.* endline x̄ = 2.25). However, the quality of preparedness for emergency C-section decreased at many hospitals (baseline x̄ = 2.4 *vs.* endline x̄ = 2.2). Overall, improvements were demonstrated in the management of obstetric complications. The main progress was seen in the management of postpartum haemorrhage, where nine hospitals improved (baseline x̄ = 1.5 *vs.* endline x̄ = 2.0), with one hospital almost meeting international standards (baseline x̄ = 1.0 *vs.* endline x̄ = 2.7) . Limited progress was seen in the management of foetal growth restriction (baseline x̄ = 0.9 *vs.* endline x̄ = 1.2) and preeclampsia, where only four hospitals slightly increased their quality of care (three made no change and three worsened). Limited improvement was observed in sepsis management, with four hospitals out of ten improving (baseline x̄ = 1.4 *vs.* x̄ = endline 1.9), two showing no difference, and four decreasing (baseline x̄ = 2.2 *vs.* endline x̄ = 1.6) (Figure S1, Panel C, Subpanels A–C in the **Online Supplementary Document**).

### Policies and organisation of care

Improvements in all three domains for policies and organisation of care could be observed for most hospitals (baseline x̄ = 1.4 *vs.* endline x̄ = 2.0). All ten hospitals showed a positive performance in managing their guidelines and audit process (baseline 1.3 *vs.* endline 2.0) (Figures S1 and S2 in the **Online Supplementary Document**).

Overall, an improvement in paediatric care could be noted, with better improvement in guidelines and audit for all hospitals (baseline x̄ = 1.3 *vs.* endline x̄ = 2.0) and less improvement in the access to and continuity of care (baseline x̄ = 1.7 *vs.* endline x̄ = 2.0).

In contrast to paediatrics, the neonatal wards considerably improved their policies in access to hospital care and continuity of care (baseline x̄ = 1.2 *vs.* endline x̄ = 2.1). Infection prevention and control (IPC) in hospital systems showed minimal improvement, and four out of ten hospitals made no changes in their IPC performance (x̄ = 1.7). The same held for the assessed maternities: IPC could be noted in four hospitals out of ten (baseline x̄ = 1.2 *vs.* endline x̄ = 1.7), and six made little difference (baseline x̄ = 1.5 *vs.* endline x̄ = 1.6) (Figure S2, Panels A–C in the **Online Supplementary Document**). One out of ten maternities achieved considerable improvements across all four organisational policy domains, with an average improvement score of 1.25 (baseline x̄ = 0.8 *vs.* endline x̄ = 1.95).

## DISCUSSION

We highlight considerable improvements in the quality of maternal, newborn, and child care across all three QI domains in the ten pilot district hospitals during the study period. Where there was little or no improvement in some hospitals, this might be attributed to interventions that mainly focused on improving clinical management capacity rather than on equipment, hospital infrastructure, or health information system support. Since our study lacked a control group, we acknowledge that it is impossible to attribute the observed improvements solely to the interventions; other factors, such as post-COVID-19 recovery, may have played a role. However, given the lack of other significant initiatives in the pilot regions during the study period, the improvements are likely due to our interventions. Studies of similar projects in African countries suggest that integrating QI approaches in a more tailored, context-specific manner has led to more sustainable health outcomes [16].

### Hospital support system

The maternal and neonatal wards showed greater improvement than the paediatric wards. Although the project did not directly support the provision of equipment or the improvement of physical infrastructure, some hospitals managed to carry out light repairs, restructure their wards, and self-procure life-saving equipment and furniture. Limited change was observed in the quality of medicine supply and laboratory services. Deterioration in drug availability and laboratory services in paediatric care was largely due to factors beyond the project’s scope, including the gradual winding down of COVID-19 emergency programmes and weak facility-level inventory management. Sustained progress in these areas will require system-wide investment and supply chain reform, not just QI measures at the hospital level. During one of the collaborative meetings, an action plan was to conduct an in-depth analysis of the availability, maintenance, and use of medical equipment. The analysis showed that hospitals lacked standardised inventory systems, functional medical devices, staff training and proper maintenance plans. Most of the equipment donated by international donors lacked regular maintenance. From personal communication with project members, following the end of the project, an earlier-established digital tool to monitor the utilisation and disposal of medical equipment is no longer available. [17].

### Policies and organisation of care

Several factors affected the differences in hospital performance. About 30% of the members in the QI committees were replaced within six months, making it harder to keep engagement and retain institutional knowledge. Hospitals that experienced changes in senior and middle management demonstrated poorer performance and staff reluctance to proactively apply the skills and knowledge they had gained. Tajikistan suffers from high medical staff turnover; the health labour market analysis reports an uneven distribution of health workers between rural and urban areas, as well as nurses with insufficient skills and training who must replace over 30% of missing doctors [18].

### Quality assurance mechanisms

Several challenges could be observed in QI efforts. Health institutions lacked sufficient capacity to conduct independent, confidential audits and relied primarily on external audits and supportive supervision visits, which evolved into regulatory visits. The system of fines and penalties observed in institutions diminishes trust and transparency and demotivates administration and staff. Similar findings from studies in sub-Saharan Africa and Asia highlight that resource limitations and punitive work environments negatively affect health service delivery [19].

A blame-free environment promotes honest reporting, open discussions, and effective learning, while fear of blame and disciplinary action often leads to inaccurate data, alienation, and harmful outcomes such as staff shortages and defensive practices [20]. In Kyrgyzstan, health workers raised concerns about the impact of such an attitude on their morale, their reluctance to work, and the lack of safeguards [21]. It is thus proposed to shift to a systems-oriented approach, replacing individual blaming with addressing systemic failures, applying a ‘No Name, No Blame, No Shame’ culture.

### IPC

IPC highlights critical aspects in preventing maternal, newborn and child morbidity and mortality, such as endometritis, sepsis, and pneumonia, causing longer unnecessary hospitalisation, misuse of antibiotics, and poor patient care [22,23]. Most facilities lacked a dedicated, trained focal person, a budget, and the resources to promote prevention of hospital-acquired infections and combat antimicrobial resistance through good IPC practices [23,24]. If sepsis is suspected, most facilities rely on external microbiology laboratories for culture and sensitivity testing. That normally takes a week to yield results, and more delays put patients’ lives at risk and add complications [24]. WHO recommends that IPC education and training should be in place for all healthcare workers, utilising team- and task-based strategies that are participatory and include bedside and simulation training, to reduce the risk of hospital-acquired infections and antimicrobial resistance [25]. The project studied hand hygiene practices across all ten hospitals, and the main gaps were compliance, infrastructure, and knowledge among health workers, as well as inconsistent water, sanitation, and hygiene, a lack of soap and alcohol gel, and limited awareness among personnel about IPC [25]. Most of the facilities had no person responsible for conducting periodic or continuous monitoring of hand hygiene practices and compliance [26]. Water was still a challenge in some facilities; most had to ration water based on the size of the water pump. Therefore, running water was not always available in all facilities [25,26]. Most facilities used dry-heat sterilisation rather than the recommended steam-heat autoclaves, resulting in improper sterilisation of reusable instruments commonly used in maternity facilities.

### Unnecessary hospitalisation

Tajikistan’s hospital system faces persistent challenges, including weak PHC, unnecessary hospitalisations, and inappropriate use of antibiotics. Low capacity of PHC workers and public trust led to excessive referrals, while informal payments encouraged long stays due to low salaries. The COVID-19 pandemic has further strained the system, disrupting service delivery and increasing hospitalisations due to respiratory illnesses [25,26]. To improve the quality and efficiency of care, PHC strengthening reforms should focus on revising hospitalisation policies, ensuring the rational use of antibiotics, and improving data management for better surveillance and decision-making. Sustained interventions are essential to reduce preventable hospitalisations and improve overall health outcomes [25,26].

### Strengths and limitations

The study had several limitations. During baseline assessment, a larger group of international and national assessors were involved, and every team was led by international experts. This could ensure a more standardised and objective scoring approach. During the endline assessment, resource constraints reduced the number of international and national assessors. Not all national assessors who participated in the baseline were the same. These could all result in bias affecting the extent of QIs. However, each team was still led by an independent, international consultant to ensure an independent assessment. The lack of control hospitals makes it impossible to attribute changes to the intervention with certainty. However, as no parallel large-scale initiatives were implemented during the study period to support the hospitals, we are confident that the improvements were indeed the result of the interventions. In addition, we conducted a parallel implementation study to independently evaluate some of the study findings and build confidence in the interventions' effects. Similar limitations are echoed in other papers [8−11].

## CONCLUSIONS

We showed that substantial improvements in the quality of maternal, newborn, and child healthcare were possible in ten pilot hospitals in Tajikistan. They were achieved through a set of complex QI interventions tailored to the findings of the baseline assessment. The project succeeded in advancing clinical management practices, hospital policies and organisational care, particularly in maternity and neonatal units. At the same time, the findings highlight ongoing challenges, including gaps in IPC, limited infrastructure, and a lack of essential equipment and supplies, which need to be addressed through system-wide approaches. These limitations highlight the importance of systemic enhancement of health systems through focused resource allocation and scaling up of achieved progress.

To enhance future interventions, QI efforts should include comprehensive support for IPC capacity and infrastructure, medical equipment, and context-specific strategies. Collaborative initiatives have already improved patient care and system efficiency, strengthening clinical practices, optimising resources, and promoting continuous learning among healthcare professionals. By fostering teamwork, data-driven decision-making, and standardised best practices, we can achieve significant improvements in patient outcomes and overall hospital performance. To maximise their impact, these initiatives – particularly collaborative QI efforts – must not only be sustained in existing hospitals but also scaled up nationwide, ensuring every healthcare facility benefits from these transformative strategies.

## Additional material


Online Supplementary Document
