# Supplementary material for: Improving the quality of care for mothers, newborns, and children in ten hospitals of the Republic of Tajikistan
Source: J Glob Health. 2026 Feb 13;16:04063. doi: 10.7189/jogh.16.04063 (PMC12903048; doi:10.7189/jogh.16.04063)
Supplement: Online Supplementary Document [file jogh-16-04063-s001.pdf]

Supplement to: Yusupova S, Rasulova G, Zakirova F, et al. Improving the quality of care for mothers, newborns, and children in ten hospitals of the Republic of Tajikistan. J Glob Health. 2026;16:04063.

HOSPITALS

Case management	A Baselin	A Endline	A Differen	B Baselin	B Endline	B Differen	C Baselin	C Endline	C Differen	D Baselin	D Endline	D Differen
**Pediatric care**												
Emergency triage and treatment	1	2.2	1.2	0	1.8	1.8	0	1.8	1.8	1.1	2.3	1.2
Case management of respiratory diseases	2	2.2	0.2	1.7	1.9	0.2	2	1.7	-0.3	2	2.3	0.3
Case management of diarrhoea	1	2.1	1.1		1.7			1.8		2.3	2.4	0.1
Case management of other conditions presenting with feve	1.2	2.1	0.9	0.6	1.8	1.2	1	1.8	0.8	1.7	2.1	0.4
Case management of anaemia and growth failure	2.2	2.3	0.1	2.2	2	-0.2	2	1.5	-0.5	2.1	2.4	0.3
Case management of chronic conditions	1.6	2.1	0.5		1.8		2.3	1.5	-0.8	1.1	1.8	0.7
Supportive care	1.8	1.9	0.1	1.8	2.1	0.3	2	1.9	-0.1	2	2.1	0.1
Monitoring and follow up	2	1.8	-0.2	2	2	0.0	2.6	1.8	-0.8	2.3	2.5	0.2
**Maternal care**												
Care for normal labour and vaginal births	1.1	2.6	1.5	0.8	2	1.2	0.9	1.7	0.8	1.8	2.3	0.5
Care for caesarean section	1.9	2.3	0.4	1.4	2.4	1.0	1.1	2.1	1.0	2.3	1.7	-0.6
Management of maternal complications	1.5	2.3	0.8	1.1	2.3	1.2	1	1.3	0.3	2	2	0
Monitoring and follow-up	1	2.8	1.8	0	1.5	1.5	1.5	2	0.5	2.3	2.2	-0.1
**Newborn care**												
Newborn infant care	1.5	2.3	0.8	1.5	2	0.5	1.4	2.1	0.7	1.7	2.1	0.4
Sick newborn care	1.1	1.9	0.8	1.2	2.2	1.0	1.3	1.9	0.6	1.6	1.8	0.2
Advanced newborn care	1	1.8	0.8	1	1.6	0.6	1	1.5	0.5		1.9	
Monitoring and follow-up	1	2.5	1.5	1	2.5	1.5	1.2	2.2	1	2	2.5	

Case management	E Baselin	E Endline	E Differen	F Baselin	F Endline	F Differen	G Baselin	G Endline	G Differen
**Pediatric care**									
Emergency triage and treatment	0.9	1.8	0.9	0.5	1.6	1.1	1.4	2.3	0.9
Case management of respiratory diseases	1.3	1.8	0.5	1	1.9	0.9	1.5	2.4	0.9
Case management of diarrhoea	2.3	1.5	-0.8	0.3	1.7	1.4	1	2.2	1.2
Case management of other conditions presenting with feve	1	1.6	0.6	0.8	1.5	0.7	1.7	2.2	0.5
Case management of anaemia and growth failure	1	1.8	0.8	1.8	2	0.2	1.1	2.3	1.2
Case management of chronic conditions	2	1.6	-0.4	0.6	1.3	0.7	1.5	1.8	0.3
Supportive care	1.4	1.9	0.5	1	1.5	0.5	1.4	2.1	0.7
Monitoring and follow up	1.4	2	0.6	1.3	2	0.7	1.2	2.2	1.0
**Maternal care**									
Care for normal labour and vaginal births	1.7	2.4	0.7	1.9	2.3	0.4	1.5	1.9	0.4
Care for caesarean section	2	2.4	0.4	1.5	2.3	0.8	2	2	0.0
Management of maternal complications	1.8	2.2	0.4	1.4	2.3	0.9	1.4	1.4	0
Monitoring and follow-up	2.3	2.3	0.0	1.7	2.5	0.8	2	2.2	0.2
**Newborn care**									
Newborn infant care	1.1	2.3	1.2	1.4	2.1	0.7	1.6	2	0.4
Sick newborn care	1	2.2	1.2	1.2	2	0.8	1.4	1.9	0.5
Advanced newborn care		2			1.7			1.5	
Monitoring and follow-up	2	2.8	0.8	1.2	2.5	1.3	2	2.2	0.2

Case management	H Baselin	H Endline	H Differen	I Baselin	I Endline	I Differen	J Baselin	J Endline	J Differen	Summary Baselin	Summary Endline	Summary Differen
**Pediatric care**												
Emergency triage and treatment	1.1	1.5	0.4	1.1	1.8	0.7	0.9	1.4	0.5	0.8	1.9	1.1
Case management of respiratory diseases	1	1.6	0.6	1	2	1.0	1	2.1	1.1	1.5	2.0	0.5
Case management of diarrhoea	1	2	1.0	1	2.2	1.2	2.3	2.3	0.0	1.4	2.0	0.6
Case management of other conditions presenting with feve	1.3	1.5	0.2	1.3	2	0.7	0.8	1.7	0.9	1.1	1.8	0.7
Case management of anaemia and growth failure	1.6	1.5	-0.1	1.6	2.2	0.6	1	2	1.0	1.7	2.0	0.3
Case management of chronic conditions	1.2	1.1	-0.1	1.2	1.8	0.6	0.9	1.7	0.8	1.4	1.7	0.3
Supportive care	1.2	1.9	0.7	1.2	2.1	0.9	0.9	1.9	1.0	1.5	1.9	0.5
Monitoring and follow up	1.2	1.8	0.6	1.2	1.9	0.7	1.3	1.8	0.5	1.7	2.0	0.3
**Maternal care**												
Care for normal labour and vaginal births	2	2	0	2	2.2	0.2	1.5	2.2	0.7	1.5	2.2	0.6
Care for caesarean section	1.4	1.4	0.0	2	2	0.0	1.8	2	0.2	1.7	2.1	0.3
Management of maternal complications	1.4	1.4	0	1.5	1.8	0.3	1.6	1.6	0	1.5	1.9	0.4
Monitoring and follow-up	2	2	0.0	2	2	0.0	2	2	0.0	1.7	2.2	0.5
**Newborn care**												
Newborn infant care	1.5	1.9	0.4	2	1.9	-0.1	1.9	2.1	0.2	1.6	2.1	0.5
Sick newborn care	1.4	1.8	0.4	1.4	1.5	0.1	1.7	1.9	0.2	1.3	1.9	0.6
Advanced newborn care		1.2			1.6		1.6	1.5	-0.1	1.2	1.6	0.5
Monitoring and follow-up	1.5	1.7	0.2	2	2.3	0.3	1.5	2.3	0.8	1.5	2.4	0.8

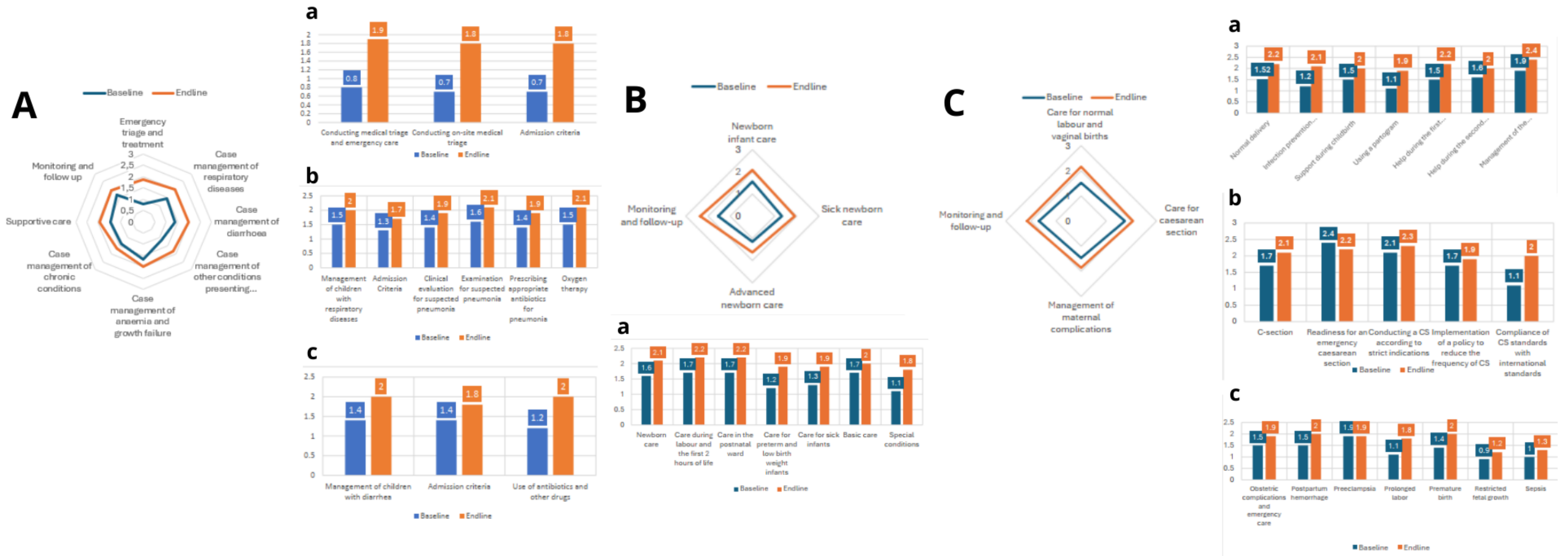

Figure S1. Heat chart for case management of baseline and endline assessment results. **Panel A.** Case management for paediatric care. Subpanel A. Paediatric care/management of patients with common illnesses. Subpanel B. Paediatric care/managing children with respiratory diseases. Subpanel C. Paediatric care/management of children with diarrhoea. **Panel B.** Case management for newborn care. Subpanel A. Newborn care. **Panel C.** Maternal care. Subpanel A. Obstetric care/normal delivery. Subpanel B. Obstetric care/C-section. Subpanel C. Obstetric care/managing obstetric complications.

HOSPITALS

Policies and organization of services	A Baselin	A Endline	A Differenc	B Baselin	B Endline	B Differenc	C Baselin	C Endline	C Difference	D Baselin	D Endline	D Differenc	E Baselin	E Endline	E Differen
**Pediatric care**															
Infection prevention	1	2.3	1.3	1.5	2.3	0.8	1.8	2	0.2	1.5	2	0.5	2.3	2.6	0.3
Guidelines and audit	1.4	2.3	0.9	1.1	2	0.9	1.5	2	0.5	1.9	2.2	0.3	1.3	2	0.7
Access to hospital care and continuity of care	2.8	2	-0.8	1.7	2	0.3	1.4	1.8	0.4	2.2	2.2	0.0	1.9	2.2	0.3
Respectful care	1.8	2	0.2	1.4	1.6	0.2	1.3	1.9	0.6	1.9	2.5	0.6	1.8	2.5	0.7
**Maternal care**															
Infection prevention	1	2.2	1.2	0.5	1.8	1.3	1.1	1.9	0.8	1.9	1.6	-0.3	1.8	1.9	0.1
Guidelines and audit	1.7	2.4	0.7	1	2.3	1.3	1.5	2.3	0.8	2.2	2.2	0.0	2.3	2.3	0.0
Access to hospital care and continuity of care	1	2	1.0	1	2	1.0	1.5	2.3	0.8	1.7	2	0.3	1.7	2.8	1.1
Respectful care	2	1.8	-0.2	0.7	1.7	1.0	1	1.8	0.8	1.3	1.8	0.5	1.3	2	0.7
**Newborn care**															
Infection prevention	1.2	2.2	1.0	1	1.8	0.8	1.5	1.9	0.4	1.8	1.5	-0.3	1.7	1.9	0.2
Guidelines and audit	1.5	2.3	0.8	1	2.3	1.3	1	2	1.0	1.6	2.2	0.6	0.9	2.3	1.4
Access to hospital care and continuity of care	1.5	2	0.5	1	2	1.0	1.5	2	0.5	0.7	2.3	1.6	0.5	2.5	2.0
Mother and newborn rights	1	2.3	1.3	1	1.7	0.7	1	1.8	0.8	2.3	2.1	-0.2	1.6	2.2	0.6

Policies and organization of services	F Baselin	F Endline	F Difference	G Baselin	G Endline	G Differenc	H Baselin	H Endline	H Differenc	I Baselin	I Endline	I Differenc	J Baselin	J Endline	J Differenc	Summary Baselin	Summary Endline	Summary Differenc
**Pediatric care**																		
Infection prevention	2.6	2.1	-0.5	1.7	1.8	0.1	1.6	1.9	0.3	1.6	1.9	0.3	1.1	1.9	0.8	1.7	2.1	0.4
Guidelines and audit	1.1	2	0.9	1.3	1.8	0.5	1.1	1.8	0.7	1.1	2.1	1.0	1.0	1.9	0.9	1.3	2.0	0.7
Access to hospital care and continuity of care	0.9	1.8	0.9	1.7	1.8	0.1	1.8	2	0.2	1.8	2.2	0.4	1.0	2.4	1.4	1.7	2.0	0.3
Respectful care	1.5	1.5	0.0	0.9	1.6	0.7	1.1	1.7	0.6	1.1	2.2	1.1	0.9	2.4	1.5	1.4	2.0	0.6
**Maternal care**																		
Infection prevention	1.2	2.4	1.2	1	1.6	0.6	2	2	0.0	1.8	1.6	-0.2	1.4	1.4	0.0	1.4	1.8	0.5
Guidelines and audit	1.2	1.9	0.7	1.5	1.6	0.1	1	1.6	0.6	1.2	1.6	0.4	1.5	1.5	0.0	1.5	2.0	0.5
Access to hospital care and continuity of care	1.5	2	0.5	1.8	2.3	0.5	1.3	2	0.7	1.5	2	0.5	2	2	0.0	1.5	2.1	0.6
Respectful care	1.5	1.7	0.2	1	1	0.0	1	1.4	0.4	1.2	2	0.8	1.7	1.8	0.1	1.3	1.7	0.4
**Newborn care**																		
Infection prevention	1.3	2.4	1.1	0.9	1.7	0.8	1.3	1.3	0.0	1.8	1.6	-0.2	1.5	1.6	0.1	1.4	1.8	0.39
Guidelines and audit	1.3	1.9	0.6	1.3	1.5	0.2	0.7	1.6	0.9	1.5	1.6	0.1	1.6	2	0.4	1.2	2.0	0.73
Access to hospital care and continuity of care	1	2	1.0	1.3	2	0.7	1.3	2	0.7	1.5	2	0.5	1.8	2	0.2	1.2	2.1	0.87
Mother and newborn rights	1.5	1.7	0.2	0.8	1	0.2	0.8	1.4	0.6	1.7	1.9	0.2	1.7	1.8	0.1	1.3	1.8	0.45

A

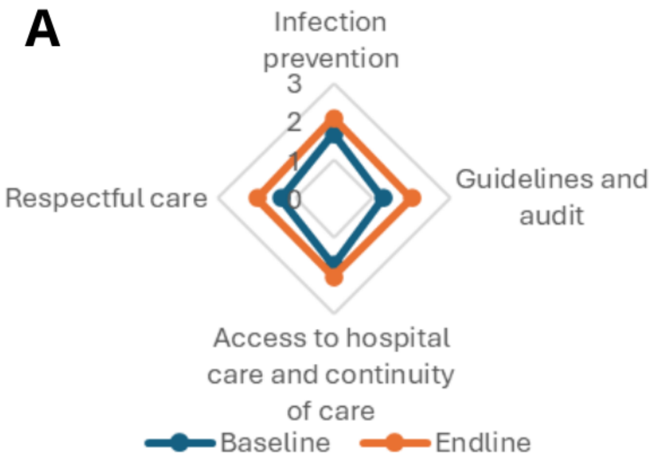

B

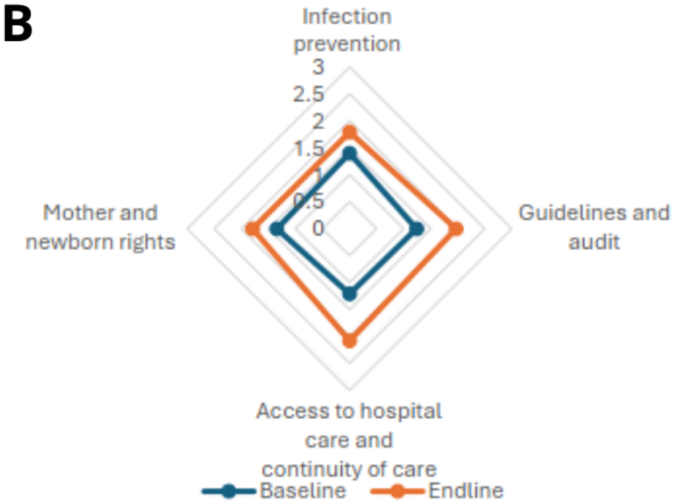

C

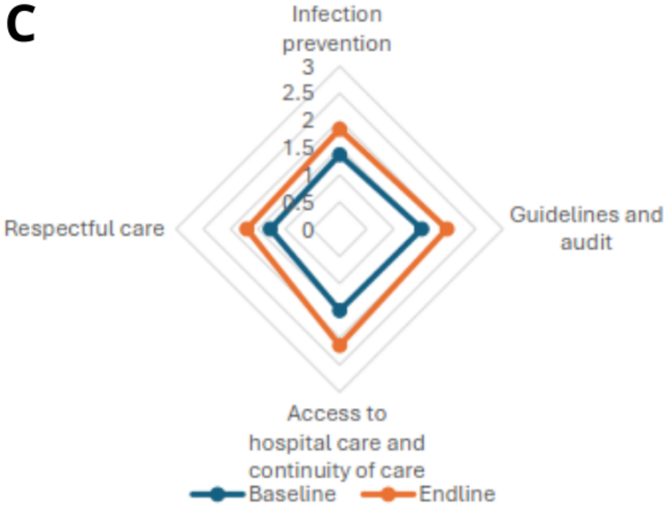

Figure S2. Heat chart for policies and organisation of care for baseline and endline assessment. **Panel A.** Policies and organisation of care in paediatrics. **Panel B.** Policies and organisation of care in newborn care. **Panel C.** Policies and organisation of care in maternal care.
